# An Experiment with Air Purifiers in Delhi during Winter 2015-2016

**DOI:** 10.1371/journal.pone.0167999

**Published:** 2016-12-15

**Authors:** Sangita Vyas, Nikhil Srivastav, Dean Spears

**Affiliations:** 1 r.i.c.e., New Delhi, India; 2 Economics and Planning Unit, Indian Statistical Institute, New Delhi, India; 3 Economics Department, University of Texas, Austin, Texas, United States; Tsinghua University, CHINA

## Abstract

Particulate pollution has important consequences for human health, and is an issue of global concern. Outdoor air pollution has become a cause for alarm in India in particular because recent data suggest that ambient pollution levels in Indian cities are some of the highest in the world. We study the number of particles between 0.5μm and 2.5μm indoors while using affordable air purifiers in the highly polluted city of Delhi. Though substantial reductions in indoor number concentrations are observed during air purifier use, indoor air quality while using an air purifier is frequently worse than in cities with moderate pollution, and often worse than levels observed even in polluted cities. When outdoor pollution levels are higher, on average, indoor pollution levels while using an air purifier are also higher. Moreover, the ratio of indoor air quality during air purifier use to two comparison measures of air quality without an air purifier are also positively correlated with outdoor pollution levels, suggesting that as ambient air quality worsens there are diminishing returns to improvements in indoor air quality during air purifier use. The findings of this study indicate that although the most affordable air purifiers currently available are associated with significant improvements in the indoor environment, they are not a replacement for public action in regions like Delhi. Although private solutions may serve as a stopgap, reducing ambient air pollution must be a public health and policy priority in any region where air pollution is as high as Delhi’s during the winter.

## Introduction

Particulate pollution has important consequences for human health [1], and is an issue of global concern. In 2014, the World Health Organization (WHO) announced that air pollution exposure represents the single largest environmental health risk, causing one-eighth of total deaths in 2012. Ambient air pollution has become a cause for alarm in India in particular because recent data suggest that ambient pollution levels in Indian cities are some of the highest in the world [2]. In fact, globally, 13 of the 20 cities with the highest mean levels of PM_2.5_ –which refers to particles with a diameter less than or equal to 2.5μm–are in India, and Delhi ranks as the worst [2].

In light of the extremely high levels of air pollution encountered in Indian cities and the risks associated with exposure, we study the number of particles of diameter between 0.5μm and 2.5μm indoors while using an air purifier in the highly polluted city of Delhi. Air purifiers are marketed as a tool that can be used to mitigate exposure to high levels of particulate pollution. However, to our knowledge, no information is available on indoor air quality during realistic patterns of air purifier use in highly polluted environments such as those found in Indian cities.

Documenting indoor air quality during air purifier use is important because of the health risks associated with particulate exposure. Exposure to PM_2.5_ has been linked to cardiopulmonary mortality [3–4] and morbidity [5] among adults, infant mortality [6], and deficits in lung function growth [7] and respiratory illness [8] among children. Because of the connection between PM_2.5_ and poor health outcomes, many countries in the world have prescribed limits for PM_2.5_ and regularly monitor its levels. However, in India, these limits are often not followed, and the health consequences are tremendous. An economic analysis of the implications India’s pollution levels have on life expectancy estimates that the more than half of Indians living in areas that do not meet the annual Indian air quality standard of 40μg/m^3^ would live on average 3.2 years longer if air pollution in these areas were reduced to the standard [9].

A number of studies have examined the impact of air purifiers on the air quality in smokers’ homes [10], on allergy and respiratory symptoms for adults and children [11–18], and on vascular health [19–21]. However, all of these studies have been conducted in cities and towns in Europe and the United States, in which pollution levels, even in the homes of smokers, are often much lower than those observed in Indian cities. A related body of literature addresses the effectiveness of air purifiers and introduces methods for determining particle removal rates attributable to air cleaning devices [22–23]. This study does not attempt to calculate improvements in air quality attributable to the air purifiers we test. Since exposure is what is most relevant for health impacts, we contribute to the literature by investigating overall indoor air quality during air purifier use, and how it responds to factors over which households or policy-makers may have some control.

In addition to mass concentrations of particulate matter, number concentrations are an important marker of air quality [24]. Although the debate is far from settled, a growing literature finds that the number concentration of ultrafine particles, meaning those smaller than 0.1μm in diameter, is more highly correlated with adverse health outcomes than mass concentration [25]. These particles are so small that they hardly contribute to particle mass, but may be even more harmful for health because they are deposited deep in the respiratory tract [25]. Studies of number concentrations in urban India are scarce. Sharma and Patel [26] estimate the number distribution from the observed mass distribution in an industrial area in Mumbai. More recently, Monkkonen et al. [27] provide direct measurements of the number concentration of particles larger than 10nm in diameter and the number size distribution of particles with diameter between 3nm and 800nm in Delhi.

This study makes two main contributions to the literature. It is the first study to our knowledge to examine indoor air quality during realistic patterns of air purifier use in a highly polluted urban environment. It is also the first to our knowledge to simultaneously document the number of particles between 0.5μm and 2.5μm in Delhi outdoors and indoors while using an air purifier.

We begin by setting the context for the study using publicly available data on mass concentrations of PM_2.5_ in Delhi made available by the US Embassy. The study was conducted over four weeks in December 2015 and January 2016, a period of time in which air pollution was reaching its highest levels over the year. We then elaborate on our research methodology. We conducted air purifier tests in a residential apartment in South Delhi. The air purifiers we tested contained high-efficiency particulate arresting filters and were two of the most affordable available. The experiment comprised six test conditions, created by crossing three filter combinations with opening and closing the door of the room every 30 minutes, or not. Data were collected using two Dylos 1700 laser particle counters, which provide minute-wise counts of particles in two size bins: 0.5μm and larger, and 2.5μm and larger.

The next section of this paper discusses the results of our experiment. Though substantial reductions in indoor number concentrations, measured as the number of particles per 0.01ft^3^, are observed during air purifier use, number concentrations are nevertheless frequently above the moderate pollution levels observed in Pittsburgh [28] and Edinburgh [29], are often above levels found in polluted areas in Germany [30], and are sometimes even above levels found in the highly polluted Yangtze River Delta in China [31]. We find that indoor air quality is worse when outdoor air quality is worse. Moreover, the ratio of indoor air quality during air purifier use to two comparison measures of air quality without an air purifier are also positively correlated with outdoor pollution levels, suggesting that as air quality worsens, there are diminishing returns to improvements in indoor air quality while using an air purifier. These results are statistically significant.

We conclude by discussing the implications of this research. The findings of this study suggest that although the most affordable air purifiers currently available are associated with significant improvements in the indoor environment, they are not a replacement for public action in regions like Delhi. Although private solutions may serve as a stopgap, reducing air pollution must be a public health and policy priority in any region where air pollution is as high as Delhi’s during the winter.

## Context

**Fig 1:**
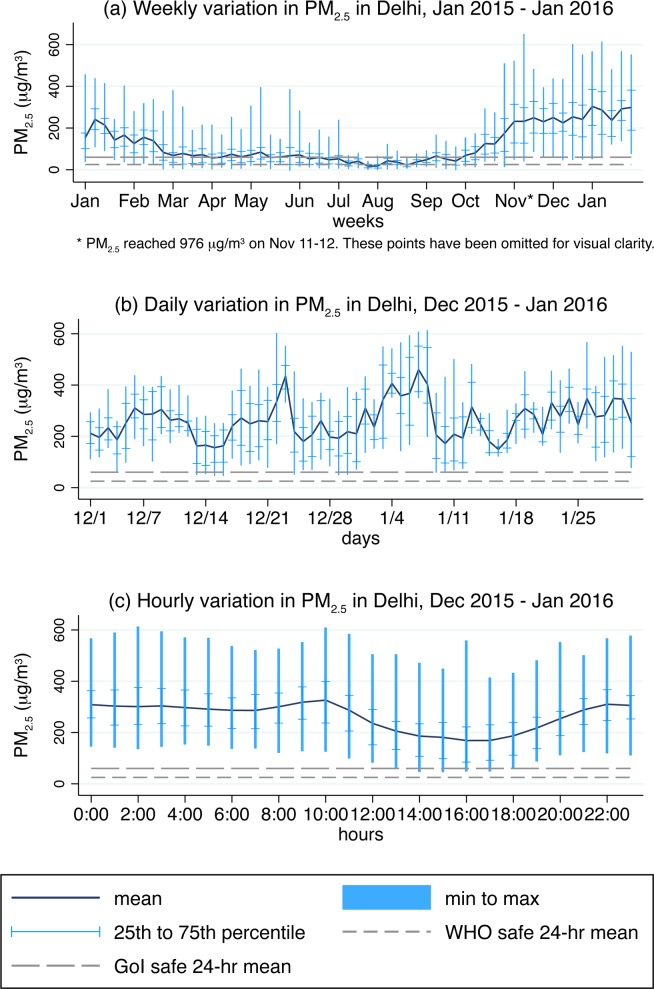
Delhi PM_2.5_ levels (μg/m^3^) regularly above international and Indian standards. Author calculations using data from the US Embassy, Chanakyapuri, New Delhi [32–33]. Means present weekly, daily, and hourly averages of hourly observations in Panels (a), (b), and (c), respectively. Similarly, minimums and maximums are displayed using bars, and 25^th^ and 75^th^ percentiles are displayed using horizontal markers, for weeks, days, and hours.

Fig 1 plots mass concentrations of PM_2.5_ in Delhi using hourly data made available by the US Embassy [32–33]. Each panel in the figure presents data over a different time window. Dark blue lines connect means, vertical bars connect minimums to maximums, and short horizontal lines indicate 25^th^ and 75^th^ percentiles. For comparison, the daily WHO limit of 25μg/m^3^ and the daily Indian government limit of 60μg/m^3^ have also been indicated in each panel. Panel (a) aggregates the hourly data by week for January 2015 through January 2016, Panel (b) aggregates by day for December 2015 through January 2016, and Panel (c) aggregates by hour for December 2015 through January 2016.

Panel (a) shows strong seasonal variation in PM_2.5_. November through February are the months with the highest concentration of PM_2.5_, March through June are characterized by PM_2.5_ levels that hover near the daily limit set by the Indian government, and July through October have the lowest levels of the year. Between January 2015 through January 2016, PM_2.5_ reached its peak reading of 976μg/m^3^ on November 12^th^ at 1:00AM, during Diwali, a holiday which is celebrated in India by setting off firecrackers. Notably, over these 13 months, the weekly mean PM_2.5_ was below the daily WHO limit of 25μg/m^3^ for only three weeks, and was below the less stringent daily Indian government limit of 60μg/m^3^ for only 17 weeks. The weekly variation in PM_2.5_ is also high, often varying over several hundreds of μg/m^3^ over the course of a week.

Panel (b) depicts daily variation in PM_2.5_ over December 2015 through January 2016, the two months during which experiments were conducted. Over this period of time, neither the daily mean PM_2.5_ nor the 25^th^ percentile ever fell as low as the Indian government limit of 60μg/m^3^. The lowest daily mean was 149μg/m^3^, the highest daily mean was 460μg/m^3^, and the mean daily mean was 260μg/m^3^ over these two months. This panel also suggests a correlation in the daily mean over time. In fact, 79% of the variation in the daily mean can be statistically explained by its first lag in a regression of daily mean PM_2.5_ on the previous day’s mean PM_2.5_.

In Panel (c), we present data from the same months as in Panel (b), but instead of daily means, we display hourly means in order to examine variation in pollution over the course of the day. PM_2.5_ remains consistently high from 10:00PM to 10:00AM, at which point it starts decreasing to reach the daily low at 4:00PM and 5:00PM. From 5:00PM to 10:00PM, it steadily increases again. The nightly mean from 10:00PM to 10:00AM is 302μg/m^3^, and the daily mean is 224μg/m^3^. Between 4:00PM and 5:00PM, mean PM_2.5_ is 169μg/m^3^. The hourly 25^th^ percentiles over these two months are never below the limit set by the Indian government.

The data presented in Fig 1 show that the period over which the experiment was conducted include the weeks with the highest mean levels of PM_2.5_ over the 13 consecutive months ending January 2016. They also show that the daily mean PM_2.5_ is highly dependent on the previous day’s pollution levels. Finally, the figure shows predictable variability in hourly pollution levels throughout the day, with higher pollution levels recorded during the night, and lower levels recorded during the day. The next section will discuss how these observations were used to design the experiment.

## Methods

### Experimenting with indoor air purifiers under realistic circumstances in Delhi

Air purifiers are marketed as a product that can reduce exposure to air pollution, and are sold in upscale electronics shops throughout Delhi. Recently, demand for air purifiers in Delhi has increased as more people have become aware of the negative health impacts of exposure to air pollution [34]. However, there is little information on indoor air quality during realistic patterns of air purifier use in highly polluted environments such as Delhi during winter.

We documented indoor particulate pollution, and the factors associated with it, during realistic patterns of air purifier use in an upper class neighborhood of Delhi during winter 2015–2016. The experiment was conducted with two of the most affordable air purifiers containing high-efficiency particulate arresting (HEPA) filters, which remove at least 99.97% of particles with a diameter of 0.3μm from the air that passes through the HEPA. Filter A is a do-it-yourself air purifier constructed by the research team by strapping a HEPA filter with dimensions 14x14x1 inches to the front of a table fan with an air flow speed of 60 cubic feet per minute. These materials cost approximately $50 all together. Filter B, costing approximately $266, is a ready-made purifier containing a fan with an air flow speed of 106 cubic feet per minute, and a HEPA filter with dimensions 9.5x15x1.25 inches. The large price differential between these two filters is a reflection of the market: ready-made air purifiers with HEPA filters available in India cost between $250 and $1,500. Although these are the most affordable purifiers available, both filters are costly relative to average monthly expenditure in urban India; the cost of Filter A is equivalent to approximately one month of inflation-adjusted per capita expenditure for the average urban Indian, while the cost of Filter B is equivalent to approximately five months [35].

Tests were conducted in a 150 square foot room in a three-bedroom apartment located in the residential neighborhood of Hauz Khas, Delhi. The three-story building in which the apartment was located was not a new construction, and as is the norm in such homes, doors and windows were not sealed with weather stripping. The room had a window unit air conditioner (AC), two large windows, and two doors, one leading to a balcony and the other to a hallway within the apartment. In order to reduce the amount of outside air entering the room, some large gaps were covered using plastic tape. Tape was only applied in two places: around the AC unit and over a hole in the glass of one of the windows.

The room in which tests were conducted was meant to be representative of bedrooms in upper class neighborhoods, with some attention paid to closing up large gaps through which outside air could penetrate. These were the gaps that could easily be closed without using special materials such as weather stripping, and without disrupting the ability to open and close doors and windows. These efforts, however, by no means sealed the room completely; there were still many gaps through which outside air could seep in, for instance between doors or windows, and walls or the floor. The tests we conducted therefore represent what can be achieved if a small amount of effort, but no additional money, is invested in sealing the room.

We recorded particulate pollution within the test room while using each filter separately. Because Filter A was relatively less expensive than Filter B, tests were conducted with one Filter A, and with two Filter As running at the same time. The cost of two Filter As was still less than half the cost of Filter B.

Although it is possible to keep doors closed for an extended period of time while one is sleeping, it is expected that room doors will be opened and closed during the day as people move around their homes. In order to understand the consequences that opening and closing doors have for air purification, we tested each filter, or set of filters, with the internal door leading to the apartment hallway closed throughout the test, and with the internal door opened and closed once every 30 minutes. Thus, there were six test conditions combining different filter options–one Filter A, two Filter As, and Filter B–with whether or not the internal door was opened and closed every 30 minutes. Each test condition was conducted 12 times. In all, we conducted 72 tests, with each test lasting three hours.

Although we undertook certain measures to ensure that the tests were representative of realistic usage, no cooking took place in the apartment during the tests, the AC unit was never switched on, the room was not cleaned while tests were taking place, and no other indoor sources of particles were introduced. The test room remained empty during the duration of the tests. For these reasons, the results from these tests reflect a best-case scenario for home use.

We conducted tests over 24 days between December 21, 2015, and January 21, 2016, the weeks in which pollution levels were reaching their highest levels. In most cases, three tests were conducted each day, between 8:00AM and 1:00AM. Because it was not possible to create multiple identical rooms, we conducted the tests consecutively in one room. This, however, introduced a problem: varying testing conditions. Outdoor air pollution is constantly changing, and if outdoor air pollution affects indoor air quality even while using a purifier, it is an important variable to control for in the experiment. If testing could have continued indefinitely, performing a simple randomization allocating one of the six test conditions to each time slot would have been sufficient to solve this problem because in the long run, average differences in outdoor pollution levels that different test conditions would have had to contend with would have approached zero. Because we did not have indefinite time to conduct the experiment, though, we employed a block randomization procedure in order to minimize the expected differences in outdoor pollution levels under which each test condition had to operate. As can be seen in Panels (b) and (c) of Fig 1, pollution levels vary significantly by time of day and day of year. Thus, we blocked our randomization over time of day and calendar day, randomly allocating six different permutations of the six test conditions.

Table 1 lists the six different permutations. Each letter stands for a test condition. The blocking method is evident from this table: each test condition is present in each row, and in each column. Because only three tests could be conducted each day, it took two days to run through one entire permutation. The order in which the six permutations were allocated was decided by randomly selecting a number from one through six, six times without replacement. The order that was generated was as follows: 6, 5, 3, 2, 1, 4. Thus, the tests associated with permutation six were conducted over the first and second days of the experiment, the tests associated with permutation five were conducted on the third and fourth days, etc. It took 12 days to complete the sequence of tests allocated through this procedure. In this way, we ensured that each test condition was conducted at least once within each time slot, and within each two-day period.

**Table 1:** Block randomization of test type by calendar day and time of day^a^.

	first day			second day		
permutation	period 1	period 2	period 3	period 4	period 5	period 6
number	12pm-3pm	4pm-7pm	10pm-1am	8am-11am	12pm-3pm	4pm-7pm
**1**	A	B	C	D	E	F
**2**	B	C	D	E	F	A
**3**	C	D	E	F	A	B
**4**	D	E	F	A	B	C
**5**	E	F	A	B	C	D
**6**	F	A	B	C	D	E

^a^ Each permutation number included an assignment for each test condition to a different time period over two days. Each test condition was thus conducted once over a two-day period. Over the course of 12 days, each test condition was also conducted once during each time period. Tests were conducted over 24 days. The first six pairs of days were randomly assigned permutation numbers one through six, without replacement. The second six pairs of days repeated the same order of permutation numbers; the only difference was that the Dylos that was used inside the first time was exchanged with the Dylos that was used outside, for each test.

After each test ended, we switched on an overhead fan, and opened a door and window in order to circulate the air in the room and return the pollutions levels inside to outdoor levels. It took approximately 15 minutes for pollution levels within the room to return to the level found outside, but in most cases, we allocated more time between tests. In most cases, tests were conducted within one hour of the assigned time slot. The electricity went out in the middle of three tests, and in those cases, we restarted the test after circulating the air in the test room, or we conducted the test again on another day at a similar time.

### Measuring particulate matter

Data on particulate matter were collected using two Dylos 1700 laser particle counters (Dylos Corporation, Riverside, CA). These monitors were chosen because of their relatively low cost yet high accuracy compared to other more expensive monitors that report particle mass [29, 36–37].

The Dylos measures particulate matter by drawing in air and particles using a small computer fan, and then funneling them past a laser beam operating at 650 nm. A photo-diode is positioned within the Dylos to capture scattered light from many different angles. According to the manufacturer, variation in flow rates are accounted for in the software used to calculate particle count.

The monitor is factory-calibrated to report particle counts in two size bins: 0.5μm and greater, and 2.5μm and greater. The monitor differentiates particle size using an algorithm performed on the measurement of scattered light. These counts represent the number of particles contained in 0.01ft^3^. Thus, we were able to calculate the number of particles of diameter between 0.5μm and 2.5μm per 0.01ft^3^ by subtracting the latter from the former. The number concentration (NC) of particles between size 0.5μm and 2.5μm per 0.01ft^3^ is thus:

$${NC}_{0.5-2.5}={NC}_{>0.5}-{NC}_{>2.5}.$$ (1)

We collected minute-wise data for each three-hour test, thus resulting in approximately 180 data points for each test. The Dylos memory has the capacity to store up to 10,000 time-stamped records. In most cases, however, we transferred the data from the monitor to a computer after each test.

In some cases during the course of the experiment, particulate pollution was so high that it exceeded the 16-bit register count of 65,536 on the display screen of the monitor. The Dylos Corporation confirmed that in these situations the register flips to zero and starts counting again. When this occurs, the count of particles greater than 0.5μm appears smaller than the count of particles greater than 2.5μm, a case which would not occur otherwise. To correct for this, whenever the display of 0.5μm was less than the display of 2.5μm, we added 65,536 to the 0.5μm count. This was the case for 176 minute-wise records, which represents 1% of the number concentration data collected outdoors.

For each test, one Dylos was placed inside the room to record indoor air quality during air purifier use, and the other Dylos was placed outside to record outdoor pollution levels. The outdoor Dylos was placed on a balcony outside an adjacent bedroom of the apartment, approximately 10 meters away from the test room on the same side of the apartment building. In order to ensure that our results were not affected by measurement variation between Dylos monitors, we randomly assigned the Dylos monitor that would be located inside for each test. Thus, for each of the 36 tests listed in Table 1, one of the two Dylos’ was randomly assigned to be inside the room. After 12 days, when the full sequence of tests was completed, the entire sequence of tests was conducted again, in the same order as before, but with the Dylos that was originally inside switched with the Dylos that was originally outside. In this way, we ensured that each Dylos was used both inside and outside for each of the 36 tests listed in Table 1.

### Are reductions in particulate matter during air purifier use sufficient?

From the health perspective, it is important to determine how much pollution remains even while using an air purifier. The data collected in this study represent the number concentration of particles of diameter between 0.5μm and 2.5μm per 0.01ft^3^. However, air quality standards are generally quoted in mass concentrations. Calculating mass concentrations from number concentrations requires assumptions on particle shape, particle density, and the size distribution of particles [36–39]. Although generally accepted assumptions on shape and density are available in the literature [39–41], the size distribution of particles is highly dependent on location and the source of pollutants. Research has been conducted on the size distribution of submicron particles in urban India, and on the size distribution in mass of particles up to 10μg, but, to our knowledge, no study has investigated the number size distribution of particles, measured in particle count, over the size range we study here.

Although it is not possible to directly compare the data collected in this experiment to air quality standards, it is possible to contextualize the number concentrations found in this experiment by comparing them to those found in other places in the world. Based on a literature review, number concentrations of particles with diameter between 0.5μm and 2.5μm were found for six other locations. As we will discuss in greater detail in the Results section, these comparisons suggest that even while using an affordable air purifier, residents of Delhi may still be exposed to high pollution levels indoors.

### What influences indoor air quality during air purifier use?

Air purifier manufacturers typically cite the Clean Air Delivery Rate (CADR), which indicates the volume of clean air produced by a purifier per minute. The speed at which clean air is produced is useful when comparing purifiers to each other, but it is less meaningful for understanding particulate matter exposure or the factors that influence indoor air quality under realistic conditions of air purifier use. Do outdoor pollution levels or opening and closing the door affect indoor air quality while an air purifier is running? These questions cannot be answered using the CADR.

Nazaroff defines air purifier effectiveness as the difference in indoor concentration during air purifier use compared to the case when no air purifier is used [42]. More recently, Macintosh et al. developed a method for differentiating between removal due to settling to surfaces and removal that can be attributed to air cleaning in particular [22]. The goal of our study, however, is not to estimate the fraction of the removal that can be attributed to the air purifier. Since exposure is what is most relevant for health impacts, we investigate overall indoor air quality during air purifier use, and how it responds to factors over which households or policy-makers may have some control. During normal air purifier use in Delhi, does indoor air quality respond to changes in outdoor air quality? Is indoor air quality affected by opening and closing the door?

We investigate how indoor air quality responds to these external factors by estimating the following linear probability model:

$${indoor\;NC}_{{0.5-2.5}_{j}}=\beta_{0}+\beta_{1}{outdoor\;NC}_{{0.5-2.5}_{j}}+\beta_{2}{door\;closed}_{j}+\beta_{3}({2\;filter\;As}_{j})+\beta_{4}{(filter\;B}_{j})+\varepsilon_{j},$$ (2)

in which *j* indexes tests. The dependent variable, *indoor NC*_*0*.*5–2*.*5*_, is the mean number concentration of particles between size 0.5μm and 2.5μm per 0.01ft^3^ indoors during test *j*, calculated using data collected at least 120 minutes after the purifier was turned on in order to allow the indoor number concentration to stabilize. The primary independent variables of interest are the first two. *Outdoor NC*_*0*.*5–2*.*5*_ indicates the mean number concentration of particles between size 0.5μm and 2.5μm per 0.01ft^3^ outdoors during test *j*, and *door closed* is a binary variable indicating whether the door stayed closed throughout the test. As control variables, we include binary indicators for the type of filter used, excluding the 1 filter A condition in order to avoid multicollinearity. These variables control for any average differences across filter types. Outdoor pollution levels are demeaned in order to facilitate the interpretation of coefficients. The constant in the model thus represents mean indoor number concentration during use of 1 Filter A, with the door opened and closed every 30 minutes, at mean outdoor pollution levels.

Indoor number concentration may be higher when outdoor number concentration is higher simply because the test started off with higher levels of particulate matter. A relevant question to investigate, then, is whether indoor air quality during air purifier use is proportionally worse to worse baseline conditions. Higher pollution outdoors, or at the beginning of the test, could correspond with proportionally higher indoor pollution while the purifier is running, or not, depending on leakages into the test room, how quickly the purifier can produce clean air, particle settling, and indoor activities that contribute to particulate pollution, including cooking, cleaning, smoking, and burning incense. Because such indoor activities did not occur during testing, indoor particulate matter depended to a large extent on leakages into the room, and how quickly the purifier could produce clean air.

To study indoor particulate pollution during purifier use relative to baseline air quality conditions, we calculate the *outcome ratio* at time *t* for test *j* as:

$${outcome\;ratio}_{tj}=\frac{indoor\;{NC}_{{0.5-2.5}_{tj}}\;(with\;purifier)}{comparison\;{NC}_{{0.5-2.5}_{tj}}\;(without\;purifier)},$$ (3)

in which *t* indexes minutes and *j* indexes tests. The numerator represents the number concentration of particles between size 0.5μm and 2.5μm per 0.01ft^3^ indoors with the purifier running, and the denominator represents a comparison number concentration of particles without the purifier running. We use two alternative measures for the denominator: outdoor particulate matter at time *t* and indoor particulate matter immediately before switching on the air purifier, at time *t* = 0. Although indoor and outdoor number concentrations differ for many reasons in addition to indoor air purifier use, it is nevertheless a useful ratio to study as it incorporates changes in testing conditions that occur after the test has begun. Using starting indoor number concentration as a baseline avoids some of the drawbacks associated with using outdoor concentration, but it is not able to capture ongoing changes in outdoor conditions. For each test, we calculate the mean *outcome ratio* by averaging over data collected at least 120 minutes after the purifier was turned on.

A useful correlation to examine is that between outdoor pollution and the *outcome ratio*. A positive relationship between outdoor pollution and the *outcome ratio* would suggest that as air quality worsens, there are diminishing returns to improvements in indoor air quality while using an air purifier. We investigate this correlation by estimating the same linear probability model as in Eq (2), replacing the dependent variable with the *outcome ratio*. We present results using both comparison measures of particulate pollution without the purifier, as described above.

## Results

The central aim of this study is to understand the quality of indoor air during realistic patterns of air purifier use in a highly polluted environment such as Delhi during the winter. We first discuss summary statistics of outdoor pollution levels during the period in which the experiment was conducted. Then, we contextualize the data from the experiment using NC_0.5–2.5_ from other urban areas, and find that even while using an affordable air purifier, Delhi residents may still be exposed to levels of particulate matter that are higher than those found in other urban areas. Finally, we investigate the factors associated with indoor air quality and the ratio of indoor air quality while using an air purifier to comparison measures of air quality without a purifier.

### Large variation in outdoor particle count over the period of the experiment

**Fig 2:**
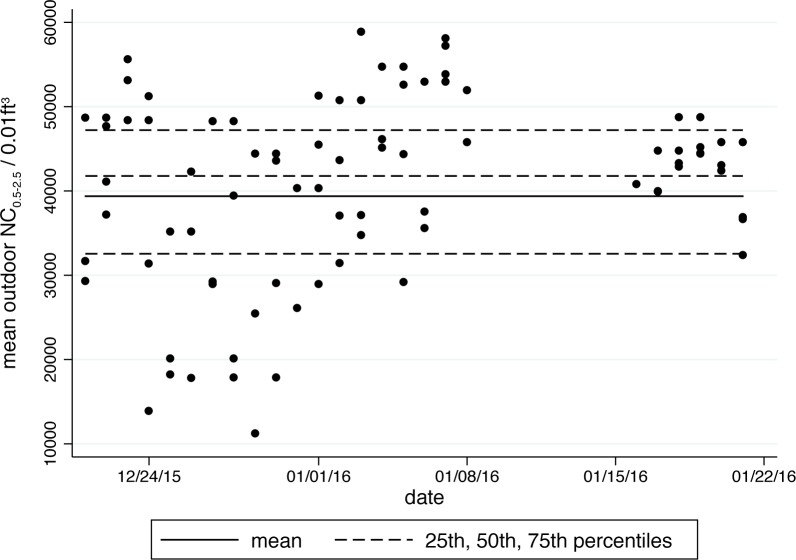
Summary statistics: Outdoor NC_0.5–2.5_ above 10,000 for all tests. Each point represents the mean outdoor NC_0.5–2.5_ during a test. 72 outdoor test means are displayed. The overall outdoor mean of all minute-wise measurements is shown as a solid line. 25^th^, 50^th^, and 75^th^ percentiles are shown as dashed lines. Outdoor NC_0.5–2.5_ were collected from a balcony near to the testing room.

Fig 2 summarizes mean outdoor NC_0.5–2.5_ by test and day. These data were collected from a balcony connected to a room that was adjacent to the test room. Number concentrations were consistently over 10,000 particles per 0.01ft^3^ over the weeks in which the experiment was conducted. The overall minute-wise mean was 39,378, and the 25^th^, 50^th^, and 75^th^ percentiles were 32,552, 41,787, and 47,219, respectively. Mean outdoor NC_0.5–2.5_ was greater than 50,000 for thirteen of the 72 tests. As can be seen from this figure, there was substantial variation in outdoor NC_0.5–2.5_ over this period of time.

In Table 2, we display overall mean outdoor NC_0.5–2.5_ by test condition. A test of equivalence suggests that the means are not statistically different from one another. This is an important result to note, as it proves that the block randomization procedure worked: outdoor air quality is not correlated with test condition.

**Table 2:** No significant difference in outdoor pollution levels across test conditions.

	outdoor NC_0.5–2.5_	standard
	per 0.01ft^3^	deviation
1 Filter A, door opened and closed every 30 mins	37,754	12,916
1 Filter A, door kept closed	40,002	14,133
2 Filter As, door opened and closed every 30 mins	38,435	11,459
2 Filter As, door kept closed	38,831	10,106
Filter B, door opened and closed every 30 mins	41,265	9,020
Filter B, door kept closed	39,957	12,157
p-value on F-test (H_0_: means not statistically		
different) = 0.97		

### NC_0.5–2.5_ while using an air purifier are often above NC_0.5–2.5_ in other outdoor urban areas

While using an air purifier, do pollution levels indoors drop below limits set by the Indian government, or the World Health Organization? Unfortunately, the Dylos data represent number concentrations, while international standards are quoted in mass concentrations. Without more information on the number size distribution of particles with diameter between 0.5μm and 2.5μm, it is not possible to convert the Dylos data to mass measurements. Instead, we compare the data from our experiment to estimates of NC_0.5–2.5_ found in the literature for other cities.

Table 3 lists mean indoor and outdoor NC_0.5–2.5_ from this study along with mean outdoor NC_0.5–2.5_ from six other cities found through a literature review. Some studies provide concentrations in particles per cm^3^, and these have been converted to the units used in this study, particles per 0.01ft^3^. Gao et al. [31] present data on number concentrations during the summer in the Yangtze River Delta, an industrial and commercial hub home to China’s largest city, Shanghai. This region is characterized by high pollution levels, and the authors note that the data collection site was situated near a large number of coal-burning power plants and factories. Moreover, the authors of the study analyzed the surface wind observations during the study period and found that emissions from Shanghai and the surrounding areas were brought to the site. Nevertheless, the mean outdoor NC_0.5–2.5_ observed in this high-pollution region in China is only 56% the outdoor NC_0.5–2.5_ we observed in Delhi. Pollution levels in China exhibit seasonality similar to that observed in Delhi, though, indicating that number concentrations from summer in China may be expected to be lower than concentrations in Delhi during the winter.

**Table 3:** Particle counts in New Delhi are much higher than in urban background sites of other cities and towns.

location	range of	mean^a^	instrument	study
(year)	particle	NC_0.05–2.5_ /	used for	author(s)
	size (μm)	0.01ft^3^	measurements	
New Delhi, indoor with air purifier	0.5–2.5	8,826	Dylos 1700	this study
New Delhi, outdoor	0.5–2.5	39,378	Dylos 1700	this study
Yangtze River Delta,	0.5–2.5	22,087^b^	Wide-range particle	Gao et al.
China (2005)			spectrometer^c^	(2009)
Zerbst, Germany	0.5–2.5	10,166 in 1993	Laser aerosol	Pitz et al.
(1993, 1999)		2,775 in 1999^d^	spectrometer^e^	(2001)
Bitterfeld, Germany	0.5–2.5	7,759 in 1993	Laser aerosol	Pitz et al.
(1993, 1999)		3,794 in 1999^d^	spectrometer^e^	(2001)
Hettstedt, Germany	0.5–2.5	6,230 in 1993	Laser aerosol	Pitz et al.
(1993, 1999)		3,256 in 1999^d^	spectrometer^e^	(2001)
Pittsburg, U.S.A.	0.5–2.5	5,264^f^	TSI aerosol particle	Stanier et al.
(2001–2002)			sizer^g^	(2004)
Edinburgh, Scotland	0.5–2.5	1,474^h^	Dylos 1700	Steinle et al.
(2012–2013)				(2015)

^a^ NC_0.5–2.5_ given in particles per cm^3^ have been converted to particles per 0.01ft^3^ by multiplying by 283.17.

^b^ Author calculations based on figures reported in Table 1 (Gao et al., 2009, pg. 830).

^c^ WPS, MSP Corporation, model 1000XP.

^d^ Author calculations based on figures reported in Table 1 (Pitz et al., 2001, pg. 4362).

^e^ PMS model LAS-X.

^f^ Author calculations based on figures reported in Table 1 (Stanier et al., 2004, pg. 3278).

^g^ TSI APS 3320 and 3321.

^h^ Author calculations of outdoor NC_0.5–2.5_ based on figures reported in Table 2 (Steinle et al., 2015, pg. 390).

In Germany, Pitz et al. [30] describe number concentrations from three counties in Eastern Germany in 1993 and 1999. The study investigates the changes in pollution levels that coincided with the reunification of Germany, after which many industries in the region shut down, domestic heating systems switched from coal to gas and oil, the car fleet was modernized, and new legal regulations regarding emissions were instituted. In two of the three counties examined in Pitz et al., mean outdoor NC_0.5–2.5_ from 1993, before all of the pollution-reducing changes had taken place, are even lower than the indoor mean we observe with an air purifier running in Delhi.

Stanier et al. [28] report on data collected in Pittsburg in 2001 and 2002 from a monitoring site set up as part of the Environmental Protection Agency’s Particulate Matter Supersites Program. The mean NC_0.5–2.5_ at this site is only 60% of the indoor mean with an air purifier running in Delhi. NC_0.5–2.5_ in Edinburgh, cited in Steinle et al. [29], is even lower. Although they were not conducted in particularly polluted areas, these two studies nevertheless provide useful comparisons of particulate matter concentrations from cities with more moderate levels of pollution.

**Fig 3:**
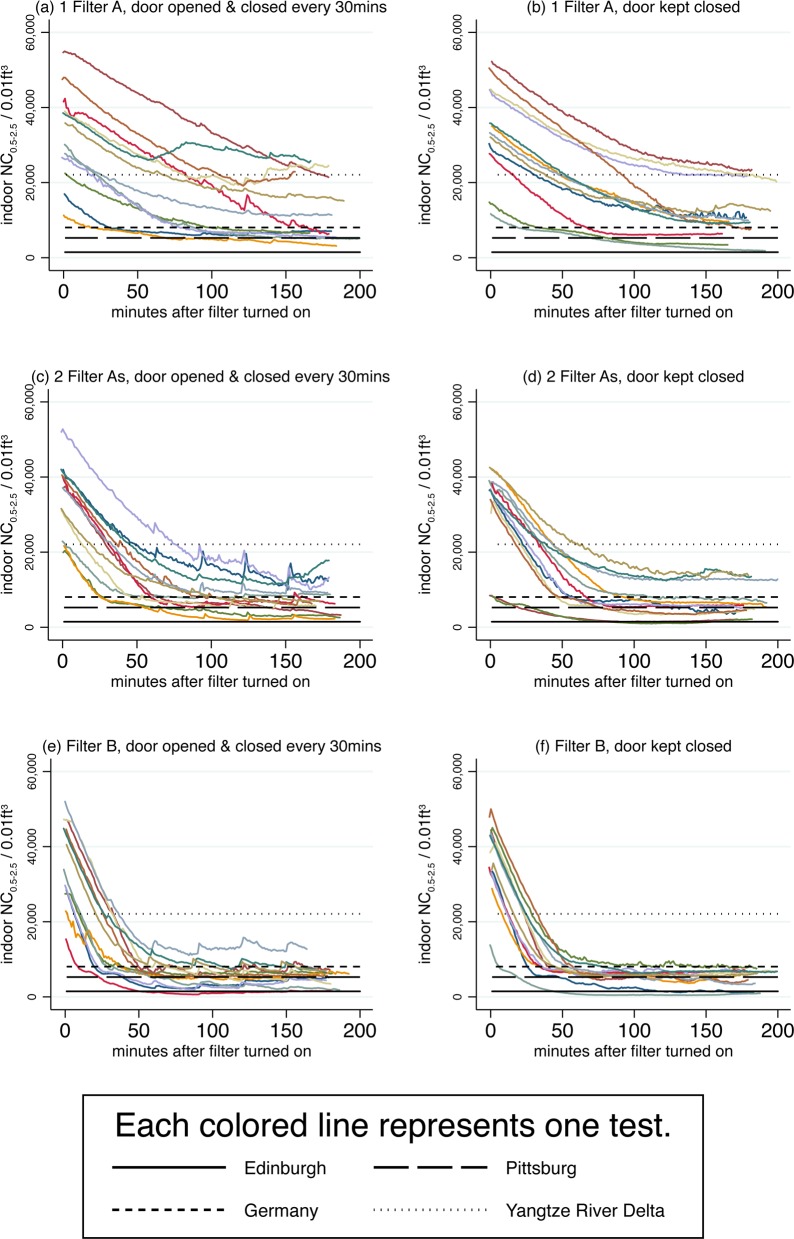
Indoor NC_0.5–2.5_ during air purifier tests often above NC_0.5–2.5_ from other urban areas. Each panel presents data from one of the six test conditions, and each test condition was performed 12 times for a total of 72 tests. Colored lines connect minute-wise NC_0.5–2.5_ reported by the Dylos. NC_0.5–2.5_ per 0.01ft^3^ for Edinburgh 2012–2013 (Steinle et al., 2015), Pittsburg 2001–2002 (Stanier et al., 2004), Germany 1993 (Pitz et al., 2001), and the Yangtze River Delta 2005 (Gao et al., 2009) are shown for comparison.

Fig 3 presents NC_0.5–2.5_ data from this study with the studies mentioned above for further comparison. Each panel in Fig 3 corresponds to one of the six test conditions, and each colored line represents all of the NC_0.5–2.5_ data from one test. Data from all 72 tests are displayed. In all panels, NC_0.5–2.5_ slopes downwards, indicating that particulate matter is reducing during air purifier use inside the test room. Horizontal black lines have been drawn at the outdoor mean NC_0.5–2.5_ for the Yangtze River Delta, Germany, Pittsburg, and Edinburgh. For simplicity, we have taken a simple average of the three measurements available from different locations in Germany. In several tests with 1 Filter A, the indoor NC_0.5–2.5_ did not even go below the NC_0.5–2.5_ in the Yangtze River Delta. Although using 2 Filter As and Filter B was always associated with pollution levels below those found in the Yangtze River Delta, particulate matter levels often exceeded levels observed in Germany in 1993 during use of 2 Filter As and often exceeded levels in Pittsburg or Edinburgh during use of Filter B. The data presented in Table 3 and Fig 3 demonstrate that during air purifier use in the Delhi winter, particulate matter indoors is frequently above the moderate levels characteristic of Pittsburgh and Edinburgh, often above levels characteristic of polluted locations in Germany, and sometimes even above levels found in a highly polluted region in China.

### Indoor air quality during air purifier use is associated with outdoor pollution levels

**Fig 4:**
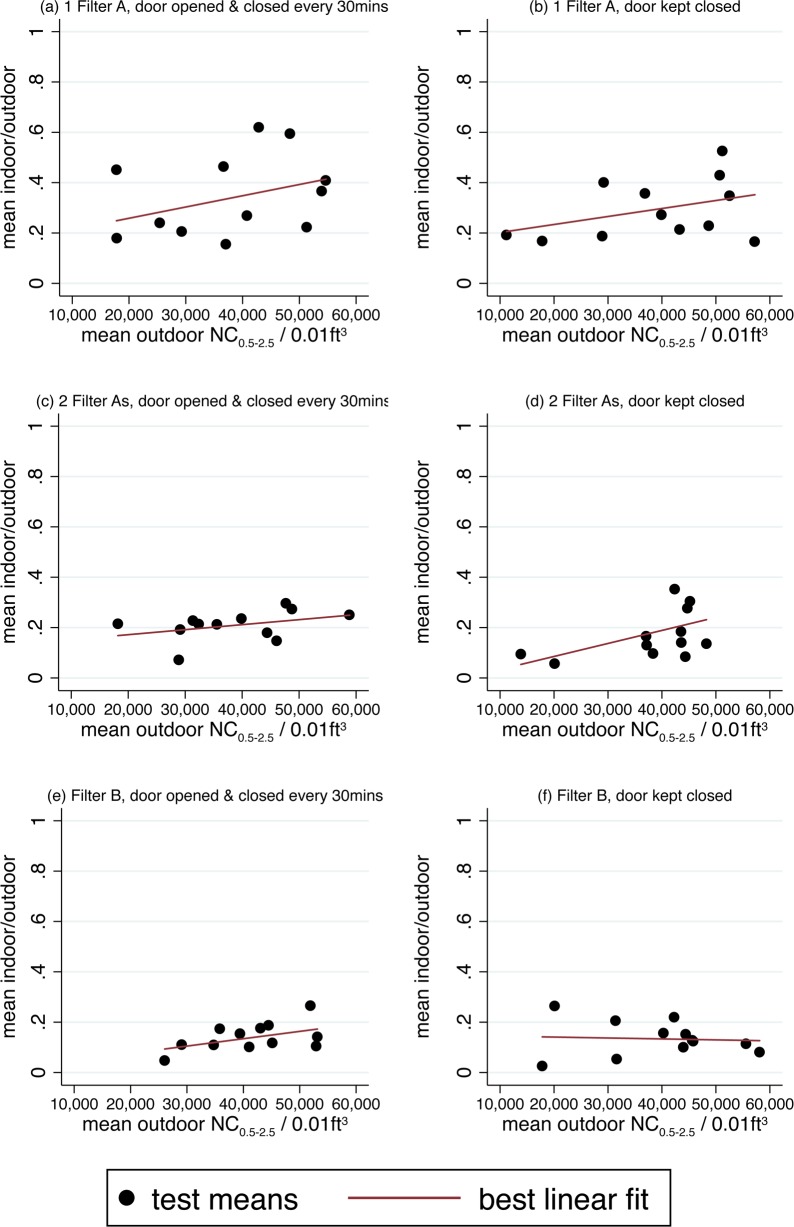
Indoor air quality depends on outdoor pollution levels. Each panel presents data from one of the six test conditions, and each dot represents the mean ratio of indoor to outdoor number concentrations during one test. Lines plot linear regressions of mean indoor-outdoor ratio on mean outdoor particle number concentration. The mean indoor-outdoor ratio for each test is calculated using data points collected at least 120 minutes after the purifier was turned on, and the mean outdoor number concentration is calculated using all of the data from the test.

Fig 4 depicts an initial result to motivate the subsequent analysis in this section: a negative relationship between outdoor pollution and the ratio of indoor to outdoor air quality, during air purifier use. There are six panels in this figure, each one corresponding to one of the six test conditions. Each panel displays test means and the best linear fit among them. In five of the six panels, the mean indoor-outdoor ratio was higher when mean outdoor NC_0.5–2.5_ was higher.

Although outdoor particulate matter is an input into the indoor-outdoor ratio, there is no mechanical relationship between outdoor number concentrations and the indoor-outdoor ratio: higher outdoor pollution could correspond with higher indoor pollution, or not, depending on leakages into the room, how quickly the purifier can produce clean air, particle settling, and indoor activities that contribute to particulate pollution. The fact that the indoor-outdoor ratio is associated with outdoor pollution suggests that as air quality worsens, there are diminishing returns to improvements in indoor air quality while using an air purifier.

Table 4 presents results from linear regressions following the specifications described in the Methods section of this paper. Each model predicts some measure of indoor air quality using outdoor pollution levels, whether the door was kept closed, and control variables. In all models, the indicator for outdoor pollution is demeaned. As control variables, both models include binary variables indicating the type of filter used, for which the filter condition of 1 Filter A is excluded in order to avoid multicollinearity. In order to allow the purifier time to stabilize pollution levels within the test room, we calculate indoor air quality means using data collected at least 120 minutes after the purifier was switched on. Means for outdoor air quality are calculated using all of the data from each test. Each model has 72 observations, one for each test, and employs robust standard errors.

**Table 4:** Linear probability models: Indoor air quality depends on outdoor pollution^a^.

	(1)	(2)	(3)
	mean indoor	mean indoor-	mean indoor-
	NC_0.5–2.5_ /	outdoor ratio^c^	starting indoor
dependent variable^b^	0.01ft^3^		ratio^d^
mean outdoor NC_0.5–2.5_ / 0.01ft^3^ during test	0.300***	0.000251**	0.000323***
(in hundreds)^e^	(0.0400)	(0.000102)	(0.0000881)
door closed	-11.48	-0.0338	-0.0382*
	(9.143)	(0.0225)	(0.0209)
constant–Filter A	135.8***	0.338***	0.384***
	(13.07)	(0.0320)	(0.0290)
2 Filter As	-53.24***	-0.130***	-0.149***
	(12.52)	(0.0308)	(0.0289)
Filter B	-77.65***	-0.187***	-0.218***
	(12.82)	(0.0311)	(0.0274)
observations (tests)	72	72	72
R-squared	0.581	0.419	0.544

^a^ Robust standard errors.

*** p<0.01

** p<0.05

* p<0.1.

^b^ Dependent variables are means calculated using data points collected at least 120 minutes after the purifier was turned on.

^c^ Mean ratio of indoor NC_0.5–2.5_ / 0.01ft^3^ at time *t* to outdoor NC_0.5–2.5_ / 0.01ft^3^ at time *t*.

^d^ Mean ratio of indoor NC_0.5–2.5_ / 0.01ft^3^ at time *t* to indoor NC_0.5–2.5_ / 0.01ft^3^ at time 0, before the filter was switched on.

^e^ Mean outdoor NC_0.5–2.5_ / 0.01ft^3^ have been demeaned for comparability of coefficients across models.

Model 1 predicts mean indoor particulate matter based on outdoor pollution levels, whether the door was kept closed, and control variables. An increase of 10,000 particles per 0.01ft^3^ outdoors, which is smaller than one standard deviation in NC_0.5–2.5_, is associated with an average increase of 3,000 particles per 0.01ft^3^ indoors. In models 2 and 3, we explore whether indoor air quality is proportionally worse when baseline conditions are worse using the outcome ratio–the ratio of indoor air quality using a purifier to a comparison measure of air quality without a purifier. Two different comparison measures of air quality are tested: model 2 uses outdoor particulate matter, and model 3 uses starting indoor particulate matter, before the filter was switched on. Both models show a statistically significant positive association between outdoor pollution levels and the outcome ratio. The coefficients suggest that an increase in NC_0.5–2.5_ per 0.01ft^3^ of 10,000 is associated with a roughly three percentage point increase in the outcome ratio.

Keeping the door closed throughout the test, versus opening and closing it every 30 minutes, is positively associated with indoor air quality. However, the coefficient on this variable is statistically significant at the 10% level only in one model. These findings do not rule out a relationship, though, as it is possible that we have too few observations to precisely estimate the association.

## Discussion

This study tested the impact of air purifiers in South Delhi during December 2015 and January 2016, a period of time characterized by very high pollution levels. The tests were conducted under conditions typical of daily life in a residential apartment representative of upper class homes in the city. Although the reductions in particulate matter associated with air purifier use were dramatic, pollution levels during this period of time were so high that indoor air quality while using a purifier was frequently worse than in outdoor areas characterized by moderate pollution, and sometimes worse than levels found in polluted locations. During air purifier use, there is a significant and large association between outdoor and indoor air pollution, suggesting that there are diminishing returns to improvements in indoor air quality as ambient air quality worsens.

The implications for health are considerable. Air purifier use is certainly associated with improvements in air quality indoors and reductions in exposure to particulate matter. However, epidemiological studies document a dose-response relationship between pollution and health, and observe health impacts from differences in pollution of even just a few micrograms per cubic meter [1]. Although we do not know the mass concentration of particulate matter inside the test room while the purifier is running, comparisons of particle count to other cities suggest that the pollution levels attained indoors during the tests we conducted are likely to still be associated with negative health impacts. Thus, although use of affordable air purifiers is associated with large reductions in particulate matter, the number of particles that remain may still be a cause for concern.

Additionally, air purifiers are only able to reduce exposure to particulate matter inside enclosed spaces, for instance in homes and offices. However, many people also spend considerable time in vehicles and public spaces, where installing an air purifier may be impractical or ineffective. Hence, affordable air purifiers provide only incomplete protection against the levels of air pollution observed in Delhi during the winter.

This study tested two of the most affordable air purifiers on the market. It is possible that other more expensive purifiers are capable of achieving lower particle number concentrations. However, considering the high cost relative to mean per capita expenditure of even the purifiers we tested, more expensive air purifiers on the market are unaffordable for an even larger fraction of the urban Indian population.

Air quality is a classic public good, indicating an important role for policy. That the most affordable private solutions provide only incomplete protection indicates that policy action is perhaps the only way to create an environment with adequately low levels of pollution for Delhi residents. In any region where this is the case, reducing air pollution must be a public health and policy priority.

## References

[1] Pope CA III, & Dockery DW. Health effects of fine particulate air pollution: lines that connect. J Air Waste Manag Assoc. 2006; 56(6): 709–742. 16805397 10.1080/10473289.2006.10464485

[2] World Health Organization. Ambient Air Pollution Database; 2014. Database. Accessed: http://www.who.int/phe/health_topics/outdoorair/databases/cities/en/.

[3] Laden F, Schwartz J, Speizer FE, & Dockery DW. Reduction in fine particulate air pollution and mortality: extended follow-up of the Harvard Six Cities study. Am J Respir Crit Care Med. 2006; 173(6): 667–672. 10.1164/rccm.200503-443OC 16424447 PMC2662950

[4] Pope CA III, Burnett RT, Thun MJ, Calle EE, Krewski D, Ito K, et al Lung cancer, cardiopulmonary mortality, and long-term exposure to fine particulate air pollution. JAMA. 2002; 287(9): 1132–1141. 11879110 10.1001/jama.287.9.1132 PMC4037163

[5] Committee on the Medical Effects of Air Pollution. Cardiovascular Disease and Air Pollution A Report by the Committee on the Medical Effects of Air Pollutant’s Cardiovascular Sub-Group. London: United Kingdom Department of Health; 2006.

[6] Chay KY, Greenstone M. The impact of air pollution on infant mortality: evidence from geographic variation in pollution shocks induced by a recession National Bureau of Economic Research 1999: No. w7442.

[7] Gauderman WJ, Avol E, Gilliland F, Vora H, Thomas D, Berhane K, et al The effect of air pollution on lung development from 10 to 18 years of age. N Engl J Med. 2004; 351(11): 1057–1067. 10.1056/NEJMoa040610 15356303

[8] Dockery DW, Speizer FE, Stram DO, Ware JH, Spengler JD, & Ferris BG Jr. Effects of inhalable particles on respiratory health of children. Am Rev Respir Dis. 1989; 139(3): 587–594. 10.1164/ajrccm/139.3.587 2923355

[9] Greenstone M, Nilekani J, Pande R, Ryan N, Sudarshan A, Sugathan A. Lower Pollution, Longer Lives. Econ Polit Wkly. 2015; 50(8): 41.

[10] Batterman S, Godwin C, & Jia C. Long duration tests of room air filters in cigarette smokers' homes. Environ Sci Technol. 2005; 39(18): 7260–7268. 16201657 10.1021/es048951q

[11] Batterman S, Du L, Mentz G, Mukherjee B, Parker E, Godwin C, et al Particulate matter concentrations in residences: an intervention study evaluating stand‐alone filters and air conditioners. Indoor Air. 2012; 22(3): 235–252. 10.1111/j.1600-0668.2011.00761.x 22145709 PMC4233141

[12] Du L, Batterman S, Parker E, Godwin C, Chin JY, O’Toole A, et al Particle concentrations and effectiveness of free-standing air filters in bedrooms of children with asthma in Detroit, Michigan. Build Environ. 2011; 46(11): 2303–2313. 10.1016/j.buildenv.2011.05.012 21874085 PMC3161201

[13] Pedroletti C, Millinger E, Dahlen B, Söderman P, & Zetterström O. Clinical effects of purified air administered to the breathing zone in allergic asthma: a double-blind randomized cross-over trial. Respir Med. 2009; 103(9): 1313–1319. 10.1016/j.rmed.2009.03.020 19443189

[14] Sulser C, Schulz G, Wagner P, Sommerfeld C, Keil T, Reich A, et al Can the use of HEPA cleaners in homes of asthmatic children and adolescents sensitized to cat and dog allergens decrease bronchial hyperresponsiveness and allergen contents in solid dust? Int Arch Allergy Immunol. 2008; 148(1): 23–30. 10.1159/000151502 18716400

[15] Francis H, Fletcher G, Anthony C, Pickering C, Oldham L, Hadley E, et al Clinical effects of air filters in homes of asthmatic adults sensitized and exposed to pet allergens. Clin Exp Allergy. 2003; 33(1): 101–105. 12534557 10.1046/j.1365-2222.2003.01570.x

[16] Van der Heide S, Kauffman HF, Dubois AE, & De Monchy JG. Allergen reduction measures in houses of allergic asthmatic patients: effects of air-cleaners and allergen-impermeable mattress covers. Eur Respir J. 1997; 10(6): 1217–1223. 9192919 10.1183/09031936.97.10061217

[17] Fisk WJ. Health benefits of particle filtration. Indoor Air. 2013; 23(5): 357–368. 10.1111/ina.12036 23397961

[18] Sublett JL. Effectiveness of air filters and air cleaners in allergic respiratory diseases: A review of the recent literature. Curr Allergy Asthma Rep. 2011; 11(5): 395–402. 10.1007/s11882-011-0208-5 21773748 PMC3165134

[19] Brauner EV, Forchhammer L, Møller P, Barregard L, Gunnarsen L, Afshari A, et al Indoor particles affect vascular function in the aged: an air filtration–based intervention study. Am J Respir Crit Care Med. 2008; 177(4): 419–425. 10.1164/rccm.200704-632OC 17932377

[20] Allen RW, Carlsten C, Karlen B, Leckie S, Eeden SV, Vedal S, et al An air filter intervention study of endothelial function among healthy adults in a woodsmoke-impacted community. Am J Respir Crit Care Med. 2011; 183(9); 1222–1230. 10.1164/rccm.201010-1572OC 21257787

[21] Weichenthal S, Mallach G, Kulka R, Black A, Wheeler A, You H, et al A randomized double‐blind crossover study of indoor air filtration and acute changes in cardiorespiratory health in a First Nations community. Indoor Air. 2013; 23(3): 175–184. 10.1111/ina.12019 23210563

[22] MacIntosh DL, Myatt TA, Ludwig JF, Baker BJ, Suh HH, & Spengler JD. Whole house particle removal and clean air delivery rates for in-duct and portable ventilation systems. J Air Waste Manag Assoc. 2008; 58(11): 1474–1482. 19044163 10.3155/1047-3289.58.11.1474

[23] Shaughnessy RJ, & Sextro RG. What is an effective portable air cleaning device? A review. J Occup Environ Hyg. 2006; 3(4): 169–181. 10.1080/15459620600580129 16531290

[24] Ruuskanen J, Tuch T, Ten Brink H, Peters A, Khlystov A, Mirme A, et al Concentrations of ultrafine, fine and PM 2.5 particles in three European cities. Atmos Environ. 2001; 35(21): 3729–3738.

[25] Wichmann HE, Spix C, Tuch T, Wölke G, Peters A, Heinrich J, et al Daily mortality and fine and ultrafine particles in Erfurt, Germany part I: role of particle number and particle mass. Res Rep Health Eff Inst. 2000; 98: 5–86. 11918089

[26] Sharma VK, & Patil RS. Size distribution of atmospheric aerosols and their source identification using factor analysis in Bombay, India. Atmos Environ Part B Urban Atmosphere. 1992; 26(1): 135–140.

[27] Mönkkönen P, Uma R, Srinivasan D, Koponen IK, Lehtinen KEJ, Hämeri K, et al Relationship and variations of aerosol number and PM 10 mass concentrations in a highly polluted urban environment—New Delhi, India. Atmos Environ. 2004; 38(3): 425–433.

[28] Stanier CO, Khlystov AY, & Pandis SN. Ambient aerosol size distributions and number concentrations measured during the Pittsburgh Air Quality Study (PAQS). Atmos Environ. 2004; 38(20): 3275–3284.

[29] Steinle S, Reis S, Sabel CE, Semple S, Twigg MM, Braban CF, et al Personal exposure monitoring of PM 2.5 in indoor and outdoor microenvironments. Sci. Total Environ. 2015; 508: 383–394. 10.1016/j.scitotenv.2014.12.003 25497678

[30] Pitz M, Kreyling WG, Hölscher B, Cyrys J, Wichmann HE, & Heinrich J. Change of the ambient particle size distribution in East Germany between 1993 and 1999. Atmos Environ. 2001; 35(25): 4357–4366.

[31] Gao J, Wang T, Zhou X, Wu W, & Wang W. Measurement of aerosol number size distributions in the Yangtze River delta in China: Formation and growth of particles under polluted conditions. Atmos Environ. 2009; 43(4): 829–836.

[32] US Embassy. Ambient Air Quality Data; 2015. Database: New Delhi and Consulates 2015 [Internet]. Accessed: https://newdelhi.usembassy.gov/airqualitydataemb/jan-nov2015.csv

[33] US Embassy. Ambient Air Quality Data; 2016. Database: New Delhi and Consulates 2016 [Internet]. Accessed: http://newdelhi.usembassy.gov/airqualitydataemb/jan-2016.csv

[34] Krishnan V, and Perappadan BS. Air purifiers sell like hot cakes. The Hindu. December 9, 2015. Available: http://www.thehindu.com/news/national/other-states/air-purifiers-sell-like-hot-cakes/article7962909.ece. Accessed April 7, 2016.

[35] National Sample Survey Organization. Key Indicators of Employment and Unemployment in India 2011–2012. NSS 68^th^ Round. New Delhi: Ministry of Statistics and Programme Implementation, Government of India; 2013.

[36] Jovašević-Stojanović M, Bartonova A, Topalović D, Lazović I, Pokrić B, & Ristovski Z. On the use of small and cheaper sensors and devices for indicative citizen-based monitoring of respirable particulate matter. Environ Pollut. 2015; 206: 696–704. 10.1016/j.envpol.2015.08.035 26342459

[37] Northcross AL, Edwards RJ, Johnson MA, Wang ZM, Zhu K, Allen T, & Smith KR. A low-cost particle counter as a realtime fine-particle mass monitor. Environ Sci Process Impacts. 2013; 15(2): 433–439. 10.1039/c2em30568b 25208708

[38] Binnig J, Meyer J, & Kasper G. Calibration of an optical particle counter to provide PM2. 5 mass for well-defined particle materials. J Aerosol Sci. 2007; 38(3): 325–332.

[39] Tittarelli A, Borgini A, Bertoldi M, De Saeger E, Ruprecht A, Stefanoni R, Tagliabue G, Contiero P. Crosignani P. Estimation of particle mass concentration in ambient air using a particle counter. Atmos Environ. 2008; 42(36): 8543–8548.

[40] Sarangi B, Aggarwal SG, Sinha D, Gupta PK. Aerosol effective density measurement using scanning mobility particle sizer and quartz crystal microbalance with the estimation of involved uncertainty. Atmos Meas Tech. 2016; 9(3): 859.

[41] Wittmaack K. Advanced evaluation of size-differential distributions of aerosol particles. J Aerosol Sci. 2002; 33(7): 1009–1025.

[42] Nazaroff WW. Effectiveness of air cleaning technologies. Proceedings of 6th International Conference of Healthy Buildings, Helsinki, Vol. 2. 2000; 49–54.

